# Oral Health Status and Patterns of Dental Service Utilization of Adolescents in Lesotho, Southern Africa

**DOI:** 10.3390/children8020120

**Published:** 2021-02-07

**Authors:** Abbas Jessani, Mir Faeq Ali Quadri, Pulane Lefoka, Abdul El-Rabbany, Kirsten Hooper, Hyun Ja Lim, Eketsang Ndobe, Mario Brondani, Denise M. Laronde

**Affiliations:** 1 Schulich School of Medicine and Dentistry, University of Western Ontario, London, ON N6A 3K7, Canada; 2 Dental Public Health, Department of Preventive Dental Sciences, College of Dentistry, Jazan University, Jizan 45142, Saudi Arabia; dr.faeq.quadri@gmail.com; 3 Faculty of Health Sciences, Nursing Department, National University of Lesotho, Maseru 180, Lesotho; pjlefoka@gmail.com; 4 College of Dentistry, University of Saskatchewan, Saskatoon, SK S7N 5E4, Canada; abdul.elrabbany@usask.ca (A.E.-R.); kirsten.hooper@usask.ca (K.H.); 5 Department of Community Health & Epidemiology College of Medicine, University of Saskatchewan, Saskatoon, SK S7N 5E4, Canada; hyun.lim@usask.ca; 6 Private Dentist, Maseru 180, Lesotho; eketsangndobe@gmail.com; 7 Oral Health Science, Faculty of Dentistry, University of British Columbia, Vancouver, BC V6T 1Z3, Canada; brondani@dentistry.ubc.ca; 8 Oral and Biological Medical Sciences, Faculty of Dentistry, University of British Columbia, Vancouver, BC V6T 1Z3, Canada; dlaronde@dentistry.ubc.ca

**Keywords:** unmet needs, Lesotho, barriers to care, dental needs, adolescents, caries, oral health, access to care

## Abstract

This study aimed to characterize the best predictors for unmet dental treatment needs and patterns of dental service utilization by adolescents in the Kingdom of Lesotho, Southern Africa. A self-reported 40-item oral health survey was administered, and clinical oral examinations were conducted in public schools in Maseru from August 10 to August 25, 2016. Associations between psychosocial factors with oral health status and dental service utilization were evaluated using simple, bivariate, and multivariate regressions. Five hundred and twenty-six survey responses and examinations were gathered. The mean age of student participants was 16.4 years of age, with a range between 12 and 19 years of age. More than two thirds (68%; *n* = 355) of participants were female. The majority reported their quality of life (84%) and general health to be good/excellent (81%). While 95% reported that oral health was very important, only 11% reported their personal dental health as excellent. Three percent reported having a regular family dentist, with the majority (85%) receiving dental care in a hospital or medical clinic setting; only 14% had seen a dental professional within the previous two years. The majority of participants did not have dental insurance (78%). Clinical examination revealed tooth decay on 30% of mandibular and maxillary molars; 65% had some form of gingivitis. In multivariate analysis, not having dental education and access to a regular dentist were the strongest predictors of not visiting a dentist within the last year. Our results suggest that access to oral health care is limited in Lesotho. Further patient oral health education and regular dental care may make an impact on this population.

## 1. Introduction

Oral health is central to general health and wellbeing [1]. This becomes particularly important when dealing with adolescents, as oral diseases can have a significant psychosocial impact and restrict daily activities, including hours lost from school and work [2]. Oral health in the most under-privileged nations is often neglected due to various psychosocial disparities such as limited resources, poverty, and lack of access to preventive dental services [3]. The countries of Southern Africa, including South Africa, Namibia, Botswana, Lesotho, and Eswatini are home to 63.4 million people. Of this population, 19.1 million are children under the age of 15 [4]. The Kingdom of Lesotho is within South Africa and has a population of 2.2 million people. Children under the age of 15 compose 35.7% of the Lesotho population [4].

The United Nations categorized Lesotho as an underdeveloped country with persistently high unemployment rates (23–28%) over the last 10 years [5]. In 10 urban centres in Lesotho, nearly a third of the population was receiving food or cash for living assistance from friends and family outside the Kingdom to provide living assistance. In the capital Maseru, 46% of the residents receive assistance in the form of food, cash, or both from outside of Lesotho, making it one of the top 20 most unequal countries in the world [6,7].

Although the government of Lesotho endeavours to provide universal primary health care for all citizens through a decentralized system, facilitating local control and decision-making at the district and community levels, Lesotho has experienced worsening health outcomes over the past decade. The World Bank Group (2018) attributed this trend largely to the burden of HIV/AIDS, comparatively high rates of tuberculosis, and systemic deficiencies [7]. Not surprisingly, preventative or therapeutic oral health care is not provided due to the shortage of oral health personnel and challenges in infrastructure [8]. A study by Umunna, James, and Ricks in 2009 indicated that the main barriers to dental care in Lesotho were shortage of oral healthcare providers and general resources compounded by transportation difficulties [9].

According to an epidemiological survey from 1998, 92% of the adult population in Lesotho had dental caries, with 93% of those receiving extractions as treatment [10]. Two decades later, a study conducted by Keating et al. (2019) with orphanages in Lesotho only reported on the number referrals to a dental professional [11]; the majority of the referrals were due to caries, with dental extraction being the most common form of treatment provided [11]. To the best of our knowledge, there is no data regarding the oral health status of the adolescent population in Lesotho. Hence, there was a need to identify psychosocial factors that impact the access to dental care and unmet dental treatment needs in adolescents in Lesotho [9], as elaborated in the framework of health service utilization proposed by Andersen and Newman (A&N) [12]. This framework categorizes the psychosocial factors into three broad categories of predisposing, enabling, and need factors. Predisposing factors include sex, the availability of a medical doctor, access to health education, having a medical condition, and water fluoridation. Enabling factors include financial affordability and means to afford dental care, including annual income, access to transportation, dental insurance, and social support. Need factors include clinical needs such as the decayed, missing, or filled teeth (DMFT) index for a given population, and subjective needs including oral hygiene, self-reported oral health, and oral health education. Using the A&N framework helps to understand the propensity of a population to access available dental services to meet their unmet dental treatment needs [13,14,15,16]. By utilizing the A&N framework of dental service utilization, this study aimed to (1) identify the unmet dental treatment needs and patterns of dental service utilization by adolescents and; (2) characterize the best predictors for perceived oral health status and dental visits for these adolescents in the Kingdom of Lesotho, Southern Africa.

## 2. Materials and Methods

Ethics approval to analyze this data was sought by the University of Saskatchewan Bio Medical Research Ethics Board (Bio-ID 650, approved 8 January 2019). This study was made possible via a collaboration between Smile Lesotho Foundation (SLF), academics from the University of British Columbia (UBC), and the National University of Lesotho (NUL) in response to Smile Lesotho Foundation’s call to identify the unmet dental treatment need of local adolescents. Faculty members and students, along with a community dentist from Maseru, educational specialists, the Minister of Health, and local stakeholders, were actively engaged in the development and execution of this project. This project was designed to serve as the foundational step in developing a program to provide long-term primary oral care to the adolescent population in that country.

### 2.1. Participant Recruitment and Data Collection

After seeking approvals from the Ministries of Health and Education and Training, participant recruitment was done through convenience sampling in the public schools located in Maseru, the capital city of Lesotho (population 2.14 million). Participants included 50 randomly selected students from each of the 10 schools from grades A/6 to grade E/12. Every fifth participant was selected from class lists provided by the school principal, until eight to twelve participants were selected form each class. Printed copies of the consent form, which outlined study objectives and sought permission to participate in the dental examination, were sent out to the parents/guardians of the participants. This study utilized a self-reported survey and clinical examinations to collect subjective and objective oral health data according to the A&N model of health service utilization. The self-reported survey was an adaptation from the Canadian Oral Health Measure Survey, and questions from World Health Organization household questionnaire were also included to capture a wide range of predictors associated with the oral health status of study participants [17,18]. The self-reported questionnaire can be found as Supplementary Materials Attachment S1. This manuscript presents only some aspects of the collected data; we have analyzed 40 items from the self-reported data, and other results will be presented in the subsequent manuscripts. The first 28 items on the survey captured the social demographic information, including the environmental risk factors. Parents/guardians responded on behalf of the participants to these questions. The last 12 items on the survey were pertinent to self-reported oral health status, including most commonly experienced dental conditions; adolescents responded to these questions, aided by volunteer nursing students from the NUL. Clinical examination followed the completion of the survey to capture the DMFT data of the study participants. These examinations were conducted in an available space either in a class room setting, a library or an open play ground. These examinations were held indoors or outdoors and utilized the chairs and writing desks. Examiners wore magnifying loupes with a head light and utilized single use disposable instruments including dental mirrors, tongue depressors, cotton rolls, and Marquis probes.

Four calibrated dentists conducted the oral examinations. Calibration exercise of the examiners involved two steps. In the first step, the clinicians were trained by the principal investigator (PI) to carry out the dental caries diagnosis through visual-tactile examination. Four permanent molars, 16, 26, 36, and 46, were examined to determine the DMFT and plaque status of the study participants. A tooth was considered to be decayed if there was a visible disruption of the enamel surface together with a tactile sense using a Marquis probe. In the second step, the readings of 10 randomly selected participants were cross checked by the PI to ensure there was consistency amongst all the examiners. A statistical analysis finding to report the calibration was ruled out because the examiners were trained by the PI, with a standard diagnosis criterion, and no discrepancies were observed in the diagnosis of the 10 randomly selected participants in pilot. All study participants, irrespective of their oral health status, were provided with oral hygiene products.

### 2.2. Statistical Analysis

Descriptive statistics were reported using numbers and percentages and then bivariate analysis was carried out using chi-square tests to identify the independent factors associated with self-reported oral health and dental visits. Univariate and multivariable logistic regression were applied to identify the most important predictors for perceived oral health and dental visits of the study sample. All univariate predictors with *p* < 0.10 were further assessed in the multivariable model. The objective of this analysis was to identify the independent variables that could strongly explain a statistically significant variation among the dependent variables in a model that is adjusted for other covariates. Adjusted odds ratio (OR) with 95% confidence interval (CI) was reported and the variables with *p*-value <0.05 were considered to be statistically significant. Statistical analysis was performed with SPSS, version 9.4 (SPSS Institute Inc., Cary, NC, USA). Missing data was replaced with the overall mean or median of that variable, although it likely reduced variance in the dataset.

## 3. Results

A total of 526 students participated in the survey and clinical examinations; not all guardians provided answers to all the demographic items. The A&N model of health service utilization served as a theoretical framework to determine the predictors that influence the unmet dental treatment needs and patterns of dental service utilization in adolescent-aged school population in Maseru, Lesotho. The findings of the univariate analyses are presented in Table 1 and Table 2; bivariate and multivariate results are presented in Table 3, Table 4 and Table 5.

Outcome variables:

The two outcome variables in this study were grouped as

1. Self-reported oral health, with (0) indicative of excellent/very good/good and (1) indicative of fair/poor;
2. Last dental visit, with (0) indicative of less than year ago and (1) one to five years ago/never.

Independent variables:

The independent variables for this study were grouped into three domains: predisposing, enabling, and needs based predictors. Predisposing factors included age, sex, and access to oral health education. Enabling factors included annual income, availability of a regular dentist and medical doctor, having dental insurance, avoidance of dental treatment due to cost, availability of social support, and availability of dental services being sought. Need factors included having clinical dental conditions such as toothaches, temperature sensitivity, bleeding when brushing, plaque status, and decay, as well as satisfaction with the overall appearance of the dentition, self-reported quality of life and general health, the importance of oral health, frequency of tooth brushing, distance to the nearest dental facility, and the reason for the last dental visit.

### 3.1. Univariate Results

The mean age of the study population was 16.4 (SD = 6.3) years, and 68% of the examined adolescents were female. The majority of the participants walked to school (88%), with only one participant travelling to school by car. Most of the participants’ parents/guardians (83%) reported that they could not afford dental insurance and that a family dentist was not available in their community (92%). Guardians reported that they lived a mean distance of 7 km from a dental facility.

Many of the adolescents reported that they have good to excellent general health (81%) and good to excellent quality of life (84%). Around 55% of the adolescents brushed their teeth twice per day; 37% brushed their teeth only once, in the morning hours. Only 32% of the participants reported consuming fluoridated water; 25% were not aware of the presence of fluoride in their drinking water. The majority (80%) of adolescents were not exposed to oral health education in school or at home (Table 1). Table 2 shows that almost one third (30%) of adolescents reported their oral health as fair or poor.

Table 2 shows that almost one third (30%) of participants reported their oral health as fair or poor. Oral health examination revealed that many of the adolescents had decay in a lower left molar (35%, tooth #36) followed by a lower right molar (30%, tooth #46). More than half of the study population had visible dental plaque.

### 3.2. Bivariate Analysis

Outcome 1: Self-reported oral health

All the predisposing, enabling, and need factors were investigated to find significant predictors for the two outcome variables. Amongst the predisposing factors, age (*p* < 0.001) was significantly associated with self-reported oral health. Participants within the age range of 12–18 were more likely to report the health of their mouth as excellent/good than the participants 19 years of age or older. The enabling factors associated with self-reported oral health included having social support (*p* = 0.02) and access to a dental office (*p* = 0.05; Table 3). The children of parents/guardians who reported having social support were more likely to rate their oral health as excellent/good. For self-reported need factors, it was observed that having a toothache (*p* < 0.001), tooth sensitivity (*p* < 0.001), and bleeding when brushing (*p* < 0.001) were significantly associated with self-reported oral health. Adolescents with no toothache, tooth sensitivity, and bleeding gum were more likely to rate the health of their mouth as excellent/good than their counterparts. Other need factors related to self-reported oral health include dissatisfaction with appearance of teeth (*p* < 0.001), general health (*p* < 0.001), and brushing frequency (*p* = 0.009). The clinical need factors included decay in teeth #16 (*p* = 0.002), #26 (*p* = 0.03), #36 (*p* = 0.003), and #46 (*p* = 0.006; Table 4).

Outcome 2: Last dental visit

The predisposing factors positively associated with a dental visit within the year included age (*p* < 0.001), sex (*p* = 0.01), and having exposure to oral health education (*p* < 0.001; Table 3). Adolescents with exposure to some form of oral health education were more likely to visit a dental professional with the last year than the adolescents who did not have such exposure. The enabling factors positively associated with having a dental visit within the last year were: availability of a regular medical doctor (*p* < 0.001), availability of a regular dentist (*p* < 0.001), avoiding dental treatment due to cost (*p* < 0.001), and where dental services were sought (*p* < 0.001). Adolescents who had access to a regular dentist and a regular medical doctor were more likely to visit a dental professional within the last year. The self-reported need factors of having a toothache (*p* < 0.001), dissatisfaction with the teeth appearance (*p* = 0.003), and having excellent/good general health (*p* = 0.007) were positively associated with the last dental visit. Of the clinical need factors, the presence of dental plaque (*p* = 0.05) and increased decay in tooth #26 (*p* = 0.001; Table 4) were associated with a dental visit within the past year.

### 3.3. Multivariate Analyses

Multivariate logistic regression (forward conditional logistic regression) was adopted to report the adjusted odds ratio and to identify the most important predictors from A&N framework for perceived oral health and last dental visit. Missing data pairwise option assisted in excluding subjects from the analyses with missing variables.

Outcome 1: Self-reported oral health

The A&N factors that best predicted perceived oral health status in adolescents are presented in Table 5. After adjusting for other predisposing, enabling, and need factors, the most important predictors for perceived oral health of adolescents were oral health education and general health. Adolescents were nearly three times more likely to report fair/poor oral health if they had no oral health education, compared to those with oral health education exposure (OR: 2.732; 95% CI: 1.144, 6.521). The odds of reporting fair/poor oral health were three times greater in study participants with self-perceived fair/poor general health in comparison to those with good/very good/excellent general health (OR:3.233; 95% CI: 1.590, 6.575).

Outcome 2: Last dental visit

The A&N factors that best predicted regularity of dental visits among the adolescents are presented in Table 5. After adjusting for other predisposing, enabling, and need factors, it was observed that oral health education, availability of a medical doctor, and increased frequency of brushing were identified as the most important predictors for regularity of dental visits. Adolescents with oral health education were four times (OR:4.559; 95%: 2.052, 10.130) more likely to visit a dentist within the last year in contrast to adolescents with access to no oral health education. In addition, availability of medical doctors within the residing area of the participants increased the odds of visiting a dentist within the last year by seven times, compared to those with no access to a regular medical doctor (OR:7.201; 95% CI: 2.273, 22.811).

**Table 1:** Descriptive data demographic variables (*N* = 526).

Independent Study Variables *	*N* (%)
Sex (*N* = 519)	
Male	164 (31.5)
Female	355 (68.4)
Age (*N* = 523)	
12–18	468 (89.5)
19+	55 (10.5)
Dental Insurance (*N* = 509)	
Yes	31 (6.1)
No	396 (77.8)
Don’t know	82 (16)
Availability of doctor (*N* = 511)	
No	469 (91.8)
Yes	42 (8.2)
Transportation to school (*N* = 319)	
Family car	1 (0.3)
Public transit	37 (11.5)
Walk	281 (87.5)
Quality of life (*N* = 518)	
Excellent	87 (16.8)
Very good	116 (22.4)
Good	231 (44.6)
Fair	75 (14.5)
Poor	9 (1.7)
Self-reported general health (*N* = 519)	
Excellent	70 (13.5)
Very good	120 (23.1)
Good	229 (44.1)
Fair	83 (16)
Poor	17 (3.3)
Self-reported brushing frequency (*N* = 506)	
Never	6 (1.2)
Only in the morning	189 (37.4)
Only before going to bed	2 (0.4)
Both times	277 (54.7)
Every time I eat	32 (6.3)
Water Fluoridation (*N* = 515)	
No	223 (43.3)
Yes	162 (31.5)
Don’t know	130 (25.2)
Self-reported last Dental Visit (*N* = 517)	
Less than a year	60 (11.6)
Between 1–2 years	13 (2.5)
Between 2–3 years	23 (4.4)
Between 3–4 years	13 (2.5)
Between 4–5 years	2 (0.4)
More than 5 years	43 (8.4)
Never	363 (70.2)
Oral health education (*N* = 518)	
No	413 (79.7)
Yes	105 (20.3)

* Response rate was less than 100% due to the missing responses.

**Table 2:** Descriptive results of oral health status of adolescents in Lesotho (*N* = 526).

Clinical Oral Health Status	*N* (%) *
DMFT tooth #16 (*N* = 524)	
Sound	380 (72.5)
Decay	141 (26.9)
Missing	3 (0.6)
Filled	0 (0)
DMFT tooth #26 (*N* = 519)	
Sound	381 (73.4)
Decay	132 (25.4)
Missing	5 (1.0)
Filled	1 (0.2)
DMFT tooth # 36 (*N* = 519)	
Sound	330 (63.6)
Decay	179 (34.5)
Missing	8 (1.5)
Filled	2 (0.4)
DMFT tooth # 46 (*N* = 521)	
Sound	352 (67.6)
Decay	157 (29.8)
Missing	10 (1.9)
Filled	2 (0.4)
Plaque status (*N* = 524)	
Absent	208 (39.7)
Present	316 (60.3)
Unhappy with appearance of teeth (*N* = 523)	
No	413 (79.0)
Yes	110 (21.0)
**Self-reported dental treatment needs**	
Toothache (*N* = 523)	
No	412 (78.8)
Yes	111 (21.2)
Sensitivity to hot/cold (*N* = 523)	
No	275 (52.6)
Yes	248 (47.4)
Bleeding when brushing (*N* = 523)	
No	314 (60.0)
Yes	209 (40.0)
Self-perceived oral health (*N* = 518)	
Excellent	59 (11.4)
Very good	106 (20.5)
Good	197 (38.0)
Fair	106 (20.5)
Poor	50 (9.7)

* Response rate was less than 100% due to missing responses.

**Table 3:** Frequency distribution of the Andersen and Newman (A&N) predisposing and enabling factors between self-reported oral health and dental visit.

	Self-Reported Oral Health				Dental Service Utilization			
	All *N* (%)	Fair/Poor *N* (%)	Excellent/Very Good/Good *N* (%)	*p*-Value	All *N* (%)	Visit within the Last Year *N* (%)	Visit more than a Year Ago *N* (%)	*p*-Value
Total	518	156 (30)	362 (70)		516	153 (30)	363 (70)	
***Predisposing factors***								
**Age**								
12–18	463 (90)	129 (28)	334 (72)	<0.001	462 (90)	125 (27)	337 (73)	<0.001
19+	54 (10)	27 (50)	27 (50)		54 (10)	28 (52)	26 (48)	
**Gender**								
Male	162 (32)	53 (33)	109 (67)	0.4	162 (32)	61 (37)	102 (63)	0.001
Female	352 (69)	102 (29)	250 (71)		352 (69)	92 (26)	258 (74)	
**Oral health education**								
No	410 (79)	129 (32)	281 (69)	0.3	410 (80)	104 (25)	306 (75)	<0.001
Yes	105 (20.3)	27 (26)	78 (74)		105 (20)	49 (48)	53 (52)	
***Factors that enable dental care***								
**Annual income**								
<1000 LSL	130 (25)	55 (78)	175 (71)	0.3	234 (45)	24 (75)	210 (73)	0.7
>1000 LSL	86 (17)	16 (23)	70 (29)		87 (17)	8 (25)	79 (27)	
**Availability of a regular MD**								
No	464 (92)	138 (30)	326 (70)	0.9	464 (92)	122 (26)	341 (74)	<0.001
Yes	33 (8)	13 (31)	20 (69)		33 (8)	27 (64)	15 (36)	
**Availability of a regular dentist**								
No	502 (97)	152 (30)	350 (70)	0.6	502 (97)	142 (28)	360 (72)	<0.001
Yes	14 (3)	3 (21)	11 (79)		13 (3)	11 (79)	3 (21)	
**Dental insurance**								
Yes	31 (7)	11 (36)	20 (65)	0.6	31 (7)	9 (31)	20 (69)	1.0
No	394 (93)	121 (31)	273 (69)		394 (93)	127 (32)	266 (68)	
**Avoiding dental treatment due to cost**								
Yes	37 (7)	9 (24)	28 (76)	0.3	36 (8)	21 (58)	15 (42)	<0.001
No	472 (93)	144 (31)	328 (70)		473 (92)	129 (27)	344 (73)	
**Having social support**								
Yes	201 (39)	1 (1)	200 (82)	0.002	276 (53)	1 (3)	275 (95)	0.6
No	45 (9)	29 (41)	16 (7)		45 (9)	30 (94)	15 (5)	

**Table 4:** Frequency distribution of the Andersen and Newman (A&N) need factors between self-reported oral health and dental visit.

	Self-Reported OH				Dental Service Utilization			
	All *N* (%)	Fair/Poor *N* = 156	Excellent/Very Good/Good *N* = 362	*p*-Value	All *N* (%)	Visit within the Last Year *N* = 153	Visit more than a Year Ago *N* = 363	*p*-Value
***Self-reported needs***								
**Toothache**								
No	407 (79)	96 (25)	311 (77)	<0.001	407 (79)	106 (26)	301 (74)	<0.001
Yes	110 (21)	60 (55)	50 (46)		110 (21)	48 (44)	61 (56)	
**Sensitivity to hold or cold**								
No	272 (53)	56 (21)	216 (79)	<0.001	272 (53)	80 (30)	189 (70)	0.1
Yes	245 (47)	100 (47)	145 (59)		245 (47)	74 (30)	173 (70)	
**Bleeding gums when brushing**								
No	312 (60)	73 (23)	239 (77)	<0.001	312 (60)	100 (32)	210 (68)	0.2
Yes	205 (40)	83 (41)	122 (60)		205 (40)	54 (26)	152 (74)	
**Unhappy with teeth appearance**								
No	410 (79)	102 (25)	308 (75)	<0.001	410 (79)	109 (27)	300 (73)	0.003
Yes	107 (21)	54 (51)	53 (50)		107 (21)	45 (42)	62 (58)	
**Quality of life**								
Excellent/Very good/Good	430 (84)	107 (25)	308 (74)	<0.001	430 (84)	130 (30)	300 (70)	0.6
Fair/Poor	83 (16)	48 (58)	35 (42)		83 (16)	22 (27)	60 (73)	
**Importance of oral health**								
Extremely important/ important	498 (99)	153 (31)	345 (69)	0.8	498 (99)	145 (29)	350 (71)	0.03
Not important	5 (1)	1 (20)	4 (40)		5 (1)	4 (80)	1 (20)	
**Self-reported general health**								
Excellent/Very good/Good	417 (81)	304 (73)	112 (27)	<0.001	417 (81)	326 (70)	138 (30)	0.007
Fair/Poor	97 (19)	57 (59)	40 (41)		97 (19)	29 (69)	13 (31)	
**Frequency of tooth brushing**								
Morning	104 (20)	17 (24)	87 (37)	0.009	108 (21)	6 (19)	102 (35)	0.099
Morning and night	197 (38)	54 (76)	143 (61)		198 (38)	23 (72)	175 (61)	
After eating/never	15 (3)	0	15 (6)		26 (5)	3 (9)	23 (8)	
**Reason for last dental visit ^1^**								
Within a year	45 (9)	5 (6.9)	21 (8.6)	0.08	(27)	4 (12)	23 (8)	0.05
Emergency	119 (23)	57 (79.2)	62 (25.4)		(108)	10 (31)	98 (34)	
Never	347 (68)	10 (13.9)	161 (66)		(186)	18 (56)	168 (58)	
***Clinical need***								
Tooth # 16								
Sound	375 (73)	97 (26)	278 (74)	**0.002**	375 (73)	106 (28)	271 (72)	0.3
Decayed	140 (27)	56 (40)	84 (60)		140 (27)	45 (33)	92 (67)	
Tooth #26								
Sound	376 (74)	276 (73)	100 (27)	**0.003**	376 (74)	93 (25)	284 (75)	**0.001**
Decayed	131 (26)	82 (63)	49 (37)		131 (26)	52 (40)	77 (60)	
Tooth # 36								
Sound	325 (65)	246 (76)	79 (24)	**0.003**	325 (65)	88 (27)	239 (73)	0.4
Decayed	178 (35)	112 (63)	66 (37)		178 (35)	54 (31)	121 (69)	
Tooth #46								
Sound	346 (69)	259 (75)	87 (25)	**0.006**	346 (69)	93 (27)	256 (73)	0.3
Decayed	157 (31)	98 (62)	59 (38)		157 (31)	48 (31)	106 (69)	
Plaque status								
No	137 (26)	34 (48)	103 (42)	0.4	140 (27)	9 (28)	131 (45)	**0.005**
Yes	179 (35)	37 (52)	142 (58)		181 (35)	23 (72)	158 (55)	

^1^ Percentage totals may not equal 100 due to rounding error.

**Table 5:** Multivariate logistic regression depicting predictors from Andersen and Newman framework.

A&N Factors	Bivariate Analysis			* Multivariate Analysis			
	Crude OR (95% CI)	*p*-Value	S.E.	Adjusted OR (95% CI)	*p*-Value	S.E.	R^2^
**Self-perceived oral health**							
No oral health education	2.414 (1.051, 5.541)	0.04	0.424	2.732 (1.144, 6.521)	0.02	0.444	0.177
Received oral health education	1						
Poor/fair general health	2.619 (1.368, 5.014)	0.004	0.331	3.233 (1.590, 6.575)	0.001	0.362	
Good/excellent general health	1						
**Last dental visit**							
No oral health education	4.381 (2.090, 9.183)	<0.001	0.378	4.559 (2.052, 10.130)	<0.001	0.407	0.045
Received oral health education	1			1			
Doctor not available	7.962 (2.780, 22.803)	<0.001	0.537	7.201 (2.273, 22.811)	0.001	0.588	
Doctor available	1			1			
Less than optimum brushing frequency	1.302 (0.889, 1.905)	0.2	0.194	1.631 (1.034, 2.575)	0.04	0.233	
Optimum frequency of brushing	1			1			

* Forward conditional method adjusted for other predisposing, enabling, and need factors.

## 4. Discussion

This is the first study to identify the self-reported oral health status and patterns of dental service utilization within the adolescent population in Lesotho. We utilized the A&N model of health service utilization to identify the strongest predictors of self-reported oral health and dental service utilization, as employed in our previous studies [14,15,16].

For predisposing factors, we found that adolescents who reported receiving some oral health education were more likely to have visited a dental professional within the last year, which corroborates the findings from Jessani et al. in 2016 and Jessani et al. in 2019 [14,15]. However, 80% of adolescents had not been exposed to any form of oral health education. This is concerning, as adolescents are in a developmental stage during which they establish lifelong habits, attitudes, and behaviours [19]. Their early knowledge and behavioural habits can substantially shape their long-term habits, including improper oral care with the consequence of increases risk for preventable oral infections including dental decay [15,20]. This was further confirmed in our multivariate analysis that identified a lack of oral health education as a major predictor of both fair/poor self-reported oral health, as well as infrequent dental visits. Therefore, volunteer nursing students were trained to provide oral health education sessions in all the visited schools. These sessions included interactive presentations regarding oral health education including proper brushing and flossing technique and healthy eating habits such as limiting consumption of sugary beverages.

Our study identified several A&N enabling factors that were significantly associated with the two outcome variables, having social support, and reporting excellent/very good/good oral health, which is similar to other studies. It has been shown that having social supports such as transportation, housing, and employment can lead to better oral health and dental service utilization [14]. This social support can result in a better quality of life that can be positively related to a better perception of oral health, as reported in this study. We also found that avoiding dental treatment due to cost was significantly associated with irregular dental visits, hence financial constraints remain the most important barrier preventing access to dental care [21]. Lack of financial affordability and unmet dental treatment needs can lead to poor oral health status [22]. In several low-income countries such as Lesotho, the cost of treating dental caries can cause an extra burden on the healthcare system. Therefore, preventive oral health programs including oral health education may substantially reduce these infections and personal cost associated with the treatment [23]. Having access to a regular medical doctor and a regular dentist were positively related to having had a dental visit within the last year, as was also found by Jessani et al. in 2020 [14]. In fact, our multivariate analysis revealed the lack of availability of medical doctors increased the odds of irregular dental visits by seven times. This indicates that barriers to access to health care are widespread across health disciplines with the challenge of insufficient health care professionals in the fields of medicine and dentistry [24] Medical care in Lesotho is provided at all three levels: national, district, and local health centers, while oral health is not currently provided at the local level due to shortages of professionals and crumbling infrastructure [8].

Our study also showed positive associations between A&N needs factors such as self-reported quality of life and general health with self-reported oral health, as discussed by others [14,16]. Adolescents who reported fair/poor oral health were more likely to also report fair/poor general health and quality of life. A study in Yemen found that people who perceived their general health as very good/excellent were also likely to perceive their oral health as very good/excellent [25]. Our results further indicated that adolescents who reported having toothache, bleeding gums, and were unhappy with the appearance of their teeth were more likely to report their oral health status as fair/poor. Similar results have been reported by David et al. in a 2006 study in Kerala, India, where self-reported oral status was found to be related to appearance of teeth and caries experience [26]. The reason for this finding might be attributed to a lack of education and preventive oral health services [26].

The clinical need factor associated with self-reported oral health status was having dental decay. Not surprisingly, adolescents were more likely to report their oral health status as fair/poor if they had decayed teeth. Tooth decay is one of the most common infections reported globally, which can affect overall well-being and the quality of life. Our study found that 35% of the adolescents had decay in a lower left molar and 30% had decay in a lower right molar. This is a concern, as the average age of our population was 16.4 years, which means their first molars have erupted less than a decade ago. Although these findings are similar to other studies, they are starkly different from countries such as Finland, where the authors found that at age 15, only 5% of participants’ first permanent molars were decayed [27]. In addition, our results showed that more than half of the study population had plaque deposits, and a very slight number were identified with restored teeth. Our results support other findings that indicate that there is a significant proportion of adolescents with unmet oral health needs and improper oral hygiene practices [28]. Such indicators can negatively affect academic performance, social development, and nutritional intake, thus impeding the well-being of adolescents [29]. Adolescents with no dental plaque and less tooth decay were more likely to report regular dental visits, which yet again confirms the association between access to care and better oral health outcomes [30].

## 5. Conclusions

This is the first descriptive cross-sectional survey and clinical assessment of the oral health of adolescents in Lesotho that identified predisposing factors, enabling factors, and needs predictors. We found several psychosocial factors that correlated with the self-reported oral health status and patterns of dental service utilization in our study population. The literature on oral health promotion strategies is heavily in favour of sustainable, culturally appropriate, and community-based initiatives. Results from this study can be utilized by oral health professionals and policy makers to provide long-term prevention-based dental care to adolescent population in the Kingdom of Lesotho.

## 6. Limitations

Our results did not come without limitations. The targeted sampling approach resulted in only a small percentage of students from each school being included in the data collection. The surveys were not completed in full by all parents/guardians, and missing data may have skewed the results. Dental decay was not examined on full dentition, which may have biased the implication of the findings. Data collection did not include the DMFS status, and no radiographic examinations were performed to confirm the extent of dental decay, which likely means that decay was underestimated, as radiographs may identify decay that the dentist cannot see on visual inspection. Despite these limitations, this initial study of provides valuable insights into the unmet oral health needs of adolescents in Lesotho.

## Supplementary Materials

The following are available online at https://www.mdpi.com/2227-9067/8/2/120/s1, Attachment S1: survey questionnaire adapted from the Canadian Health Measure Survey, 2009.
