# Supplementary material for: Oral Health Status and Patterns of Dental Service Utilization of Adolescents in Lesotho, Southern Africa
Source: Children (Basel). 2021 Feb 7;8(2):120. doi: 10.3390/children8020120 (PMC7915077; doi:10.3390/children8020120)
Supplement: Supplementary file 1 [file children-08-00120-s001.pdf]

**Attachment 1: Survey Questionnaire: Oral health care needs of adolescents in the Kingdom of Lesotho**

Please take a few minutes to fill out this survey on your oral health status and practices. **Your answers and all the shared information will be kept confidential**. We also welcome your feedback and comments. Thank you for your time and participation.

### Section A: General Information

1. The patient is an: ☐ Adult ☐ Child

2. Name of Guardian (if applicable): ________________________________________

3. Personal Information

| | | | |
|---|---|---|---|
| Number by school: | | Gender | Age |
| School Mailing Address | City | District | Postal Code |
| Email | Phone | | |

4. Where do you live (Name of village): __________________________________

### Section B: General Health

The following section includes questions about your general health. This will help us analyze any potential relationship of your self-reported general health to your self-perceived dental health. From the following questions, please select the answer that best represents your current situation:

5. In general, how would you rate your quality of life? (Please choose one)

☐ Excellent ☐ Very good ☐ Good ☐ Fair ☐ Poor

6. In general, how would you rate your general health? (Please choose one)

☐ Excellent ☐ Very good ☐ Good ☐ Fair ☐ Poor

7. Do you have a regular medical doctor?

☐ Yes ☐ No

8. Have you experienced any medical condition?

☐ Yes ☐ No

**If yes**, please name the illness: ___________

9. Are you currently on any medications?

☐ Yes ☐ No

**If yes**, please provide the names of your past and present medications:

**Section C: Oral Health**

The following section includes questions about your oral health. This will help us identify the self-reported dental health status, common self-perceived dental conditions, self-reported treatment needs and frequency of dental visit. From the following questions, please select the answer that best represents your current situation:

10. How would you rank the dental health of your family? (Please choose one)

☐ Excellent ☐ Very good ☐ Good ☐ Fair ☐ Poor

11. In general, how would you rate the health in your mouth? (Please choose one)

☐ Excellent ☐ Very good ☐ Good ☐ Fair ☐ Poor

12. How important do you think the health of your mouth is? (Please choose one)

☐ Very important ☐ somewhat important ☐ Not important
☐ Not at all important ☐ I don't know

13. How important do you consider dental health to be for your family?

☐ Very important ☐ somewhat important ☐ Not important
☐ Not at all important ☐ I don't know

14. How often do you brush your teeth?

☐ Never ☐ In the morning ☐ before bed
☐ Twice day (includes and morning before bed) ☐ Every time I eat

15. How often do you and children in your household brush your teeth?

☐ Never ☐ In the morning ☐ Before bed
☐ Twice day (includes and morning before bed) ☐ Every time I eat

16. Among children in your household, what method is largely used to clean teeth?

☐ Brush/paste ☐Dental floss ☐ Water only ☐ Traditional instrument ☐ Other

17. Does your water supply contain fluoride?

☐ Yes ☐ No ☐ Do not know

18. Have you or any family experienced any form of dental health education?

☐ Yes ☐ No

19. If so where/when? ______________________________________________

20. Where do family members go for dental health problems?

☐Nowhere ☐ Dentist ☐ Nearby clinic ☐ Hospital ☐Other

21. How many kilometers is the closest dental care facility? ______________________________

22. Do you have a regular dentist?

☐ Yes ☐ No

23. If you went to a dentist for treatment tomorrow, how would you feel?1

☐ Not anxious ☐ Slightly anxious ☐ Fairly anxious ☐ Very anxious

24. When was the last time you saw a dental professional (dentist or dental hygienist)? (Please choose one)

☐ Less than 1 year ago ☐ 1 year to less than 2 years ago
☐ 2 years to less than 3 years ago ☐ 3 years to less than 4 years ago
☐ 4 years to less than 5 years ago ☐ 5 or more years ago
☐ Never

25. When was the last time an adult in the family last saw a dental professional (dentist or dental hygienist)? (Please choose one)

☐ Less than 1 year ago ☐ 1 year to less than 2 years ago
☐ 2 years to less than 3 years ago ☐ 3 years to less than 4 years ago
☐ 4 years to less than 5 years ago ☐ 5 or more years ago
☐ Never

26. How often do you see a dental professional (dentist or dental hygienist)? (Please choose one).

☐ More than once a year for checkups or treatments
☐ About once a year for checkup or treatment
☐ Less than once a year for checkup or treatment
☐ Only for emergency care ☐ Never

27. Have you ever felt discriminated against by a dental professional because of your condition? (If applicable)

☐ Yes ☐ No Comments

28. Have you or anyone in your family experienced any difficulty in accessing dental healthcare when needed?

☐ Yes ☐ No

If your answer is no, what were the reasons

☐ Dentist unwilling to provide care ☐ financial cost ☐ too far/transportation problem
☐ Afraid of dentist ☐ Not a priority ☐ other

29. In the past 12 months, have you avoided having some or all the dental treatment that was recommended because of the cost

☐ Yes, because I could not afford the cost of treatment ☐ No ☐Other

30. Do you presently experience one or more of the following conditions. (You may choose more than one answer)

☐Toothache
☐ Sensitivity in your teeth when consuming something hot or cold
☐ Pain around your jaw joints
☐ Bleeding gums while brushing your teeth
☐ Persistent dry mouth
☐ Persistent bad breath
☐ White patch on your tongue
☐ Hole in teeth (decay)
☐ Persistent ulcers
☐ Excessive bleeding gums
☐ Loosening of teeth
☐ Swelling around neck
☐ Severe mouth pain at night
☐ Severe tooth pain at night
☐ I have pains in my jaw(s)
☐ I am not happy with the appearance of my teeth
☐ Other

31. Do you think you have any untreated condition(s) in your mouth?

☐Yes ☐ No ☐ I don't know

Comments

---

32. **If yes**, then what management of these untreated conditions do you think you need? (You may choose more than one answer)

☐ I need cleaning
☐ I need filing(s)
☐ I need root canal treatment
☐ I need orthodontic (braces) treatment
☐ I need partial denture
☐ I need full denture
☐ I need implant, bridge or crown
☐ I don't know
☐ Other

33. Are you aware of the effects of bad oral hygiene?

☐ Yes | ☐ No

34. What is the prime reason for lack of dental care? (Select all that apply)

☐ Too Expensive ☐ Too far ☐ Not enough dentists ☐ Awareness ☐ Quality of care ☐ Other

35. How willing are you to travel for dental treatment?

☑ Up to 10km ☐ 10 km - 20km ☐ To Maseru city

36. How often would you visit a dentist?

☐ Once a year ☐ Twice a year

☐ As required by dentist

37. On an average how much have you paid for a dental visit?

______________________

38. How much are you willing to pay for a dental visit?

☐ 50 LSL -75 LSL ☐ 75 LSL – 100 LSL ☐ 100 LSL – 200 LSL

39. Have you been recommended dental treatment in the last 2 years?

☐ Yes | ☐ No | ☐ Don’t know

40. Any additional feedback or concerns.

______________________________________________

______________________________________________

______________________________________________

______________________________________________

Thank you for taking the time to fill out our survey. Your participation is greatly appreciated.
